# Modern Contraceptive and Dual Method Use among HIV-Infected Women in Lusaka, Zambia

**DOI:** 10.1155/2011/261453

**Published:** 2011-10-05

**Authors:** Carla J. Chibwesha, Michelle S. Li, Christine K. Matoba, Reuben K. Mbewe, Benjamin H. Chi, Jeffrey S. A. Stringer, Elizabeth M. Stringer

**Affiliations:** ^1^University of Alabama School of Medicine, 1530 Third Avenue South, CRWH-379, Birmingham, AL 35294, USA; ^2^Centre for Infectious Disease Research in Zambia, P.O. Box 34681, Lusaka 10101, Zambia; ^3^Zambian Ministry of Health, Ndeke House, Lusaka 10101, Zambia

## Abstract

HIV-infected women in sub-Saharan Africa are at substantial risk of unintended pregnancy and sexually transmitted infections (STIs). Linkages between HIV and reproductive health services are advocated. We describe implementation of a reproductive health counseling intervention in 16 HIV clinics in Lusaka, Zambia. Between November 2009 and November 2010, 18,407 women on antiretroviral treatment (ART) were counseled. The median age was 34.6 years (interquartile range (IQR): 29.9–39.7), and 60.1% of women were married. The median CD4^+^ cell count was 394 cells/uL (IQR: 256–558). Of the women counseled, 10,904 (59.2%) reported current modern contraceptive use. Among contraceptive users, only 17.7% reported dual method use. After counseling, 737 of 7,503 women not previously using modern contraception desired family planning referrals, and 61.6% of these women successfully accessed services within 90 days. Unmet contraceptive need remains high among HIV-infected women. Additional efforts are needed to promote reproductive health, particularly dual method use.

## 1. Introduction

The Millennium Development Goals, adopted in New York in 2000, promote universal education and gender equality, maternal and child health, and prevention and treatment for HIV/AIDS [1]. Provision of comprehensive reproductive health care is central to attaining these goals [2]. Worldwide, as many as one-third of the 357,000 annual maternal deaths are attributable to unintended pregnancies; the majority of these mortalities occur in low- and middle-income countries [3–6]. Enhanced access to family planning services in sub-Saharan Africa would result in marked reductions in unintended pregnancies and unsafe abortions and a projected 69% decrease in maternal deaths and a 57% decrease in newborn deaths [4]. In addition to substantial risks of dying from pregnancy complications [7], women in a sub-Saharan Africa are also at increased risk of HIV and other sexually transmitted infections [8, 9]. Providing safe, effective contraception to HIV-infected women who desire it has also been identified by the World Health Organization as a primary strategy for prevention of pediatric infections [10]. 

In Zambia, as in many other sub-Saharan African countries, HIV-infected women of childbearing age represent a vulnerable population. The burden of STIs is high among HIV-infected pregnant women in this setting [11]. Additionally, only 20–40% of HIV-infected Zambian couples, whether serodiscordant or concordant, report use of a modern contraceptive method other than condoms [12, 13]. Public health programs that emphasize dual family planning methods—highly effective modern contraception coupled with condom use—will ensure protection from both unintended pregnancy, and STIs, and should form the cornerstone of reproductive health care. In HIV care and treatment programs, medication adherence counseling provides a unique window of opportunity to address preventive health recommendations, including family planning and STI prevention. In this paper, we describe implementation of an integrated, reproductive health peer counselor program in 16 public-sector HIV clinics in Lusaka, Zambia. We also report (1) baseline modern contraceptive and dual method use among HIV-infected women receiving antiretroviral treatment, (2) uptake of modern contraceptive and dual methods following peer counseling, and (3) predictors of modern contraceptive and dual method use among HIV-infected women.

## 2. Materials and Methods

Lusaka is home to almost 2 million people [14]. The HIV prevalence rate among pregnant women is 21% [15], and the majority of women infected with HIV are of reproductive age. The Ministry of Health's ART program was established in 2004 and covers the entire city. Over 100,000 HIV-infected individuals in Lusaka are now receiving care in this system. Due to the large volumes of patients and human resource shortages [16], peer educators conduct most ART counseling sessions. Prior to our intervention, neither routine family planning nor dual method counseling was provided during peer counseling sessions. To expand the scope and effectiveness of these counseling visits, we designed and implemented a reproductive health peer counselor program integrated within 16 primary care HIV clinics in Lusaka. 

We trained 109 peer counselors to deliver a standardized counseling message, emphasizing dual methods. The counseling intervention was implemented within the context of routine clinical care. With the aid of a printed counseling tool, peer educators delivered a comprehensive reproductive health message, including information on the range of barrier methods, hormonal and intrauterine contraception, and permanent sterilization. Women who desired access to reproductive health services were referred to a separate, on-site family planning department. In order to support public-sector reproductive health service provision, we also trained 42 family planning nurses. Training was based on the national family planning curriculum and nurses who successfully completed both a classroom-based course and a mentored, clinical practicum-received certification. Public-sector family planning clinics provided condoms, oral contraceptive pills (OCPs), depot medroxyprogesterone acetate (DMPA), Jadelle levonorgestrel implants, and copper intrauterine devices (IUDs). Women who wished to undergo permanent sterilization were referred to a center with surgical facilities, such as the University Teaching Hospital. 

Our analysis cohort included HIV-infected women aged 16–50 years and on ART at one of the 16 intervention clinics. To be eligible, a woman had to have at least one reproductive health counseling visit documented in her medical record between November 2009 and November 2010. We report baseline sociodemographic data, CD4^+^ cell count (cells/uL), hemoglobin level (g/dL), and history of tuberculosis. These data were ascertained through review of women's electronic medical and laboratory records. Laboratory results were assessed within 90 days of the woman's counseling visit. We also report use of modern contraception, including dual method use. Peer counselors collected data relating to reproductive health counseling and contraceptive use on a clinical form developed for the project. We considered condoms, OCPs, DMPA, Jadelle, IUDs, and sterilization as modern contraception. Dual method use was defined as use of condoms to prevent STIs coupled with use of a short- or long-term reversible contraceptive or sterilization. Contraceptive use data were self-reported. 

Univariate and multivariate regression analyses were used to identify sociodemographic and other predictors independently associated with modern contraceptive and dual method use, as well as with access to family planning services within 90 days of a counseling visit. Crude odds ratios (ORs) and 95% confidence intervals (CIs) were computed using logistic regression models. Adjusted odds ratios (AORs) and their corresponding 95% CIs were generated using generalized estimating equations to account for clustering at the site level. All statistical analyses were performed using SAS version 9.1.3 (SAS Institute Inc, Cary, North Carolina). Ethical approval for this study was obtained from the University of Zambia Biomedical Research Ethics Committee (Lusaka, Zambia) and the University of Alabama at Birmingham Institutional Review Board (Birmingham, AL, USA).

## 3. Results

Between November 2009 and November 2010, 32,998 women had a least one clinic visit at a site where the counseling program had been implemented. Project staffing levels had been calculated on an estimated 20,000 clinical visits during the one-year implementation period. Therefore, for logistic reasons, not all women accessing HIV treatment services at participating clinics received reproductive health counseling. Over the study period, documented reproductive health counseling visits were available for 18,407 (55.8%) HIV-infected women. Baseline characteristics of women who completed at least one counseling visit compared with those who did not receive reproductive health counseling are detailed in Table 1 . With the exception of median CD4^+^ cell count and median treatment duration, differences between women who received the counseling intervention and those who did not were not clinically significant. 

### 3.1. Cohort Description

Our analysis cohort included 18,407 HIV-infected women. At enrolment, the median age was 34.6 years (IQR: 29.9–39.7 years), 60.1% of women were married, 87.4% had one or more living children, and 39.2% had completed some secondary education. 63.0% of women reported their monthly income as ≥200,000 Zambian kwacha (approximately $45). The median CD4^+^ cell count was 394 cells/uL (IQR: 256–558 cells/uL), and the median hemoglobin level was 12.4 g/dL (IQR: 11.3–13.4 g/dL). The median time on ART was 709 days (IQR: 262–1,302 days). 3,441 (18.7%) women reported a history of or current active tuberculosis infection. Less than half (42.5%) of the women in our cohort had disclosed their serostatus to a partner. Furthermore, most women (77.1%) did not know or did not provide information about their partner's HIV status at the time of enrollment into care.

### 3.2. Baseline Modern Contraceptive and Dual Method Use

**Figure 1:**
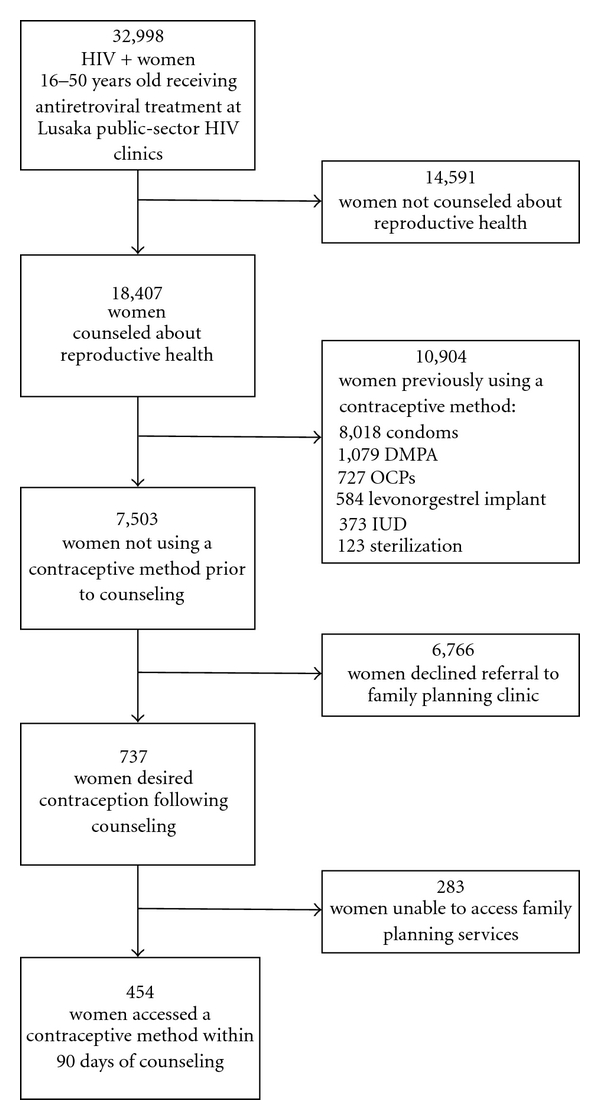
Description of the study cohort.

Of the 18,407 women included in the analysis, 10,904 (59.2%) reported current use of a modern contraceptive method: 73.5% of women reported condom use, 9.9% DMPA use, 6.7% OCP use, 5.4% levonorgestrel implant use, 3.4% IUD use, and 1.1% had undergone permanent sterilization (Figure 1). Among the 10,904 women who reported use of a modern contraceptive method at their counseling visit, 1,927 (17.7%) stated that they used dual methods for both pregnancy and STI prevention.

### 3.3. Impact of the Counseling Intervention and Unmet Contraceptive Need

Of the 7,503 (40.8%) women in our cohort not using modern contraception, 737 (9.8%) women desired contraception after counseling and 71 stated an intention to use dual methods. 454 of 737 (61.6%) women who desired contraception successfully accessed family planning services within 90 days of their counseling visit. Our data, therefore, indicate that nearly 40% of women who desired reproductive health services were unable to access public-sector services (i.e., free from point-of-service user fees). This represents substantial unmet contraceptive need within Lusaka's public health system. 

In univariate analysis, age was the only factor associated with successful access to contraceptive services within 90 days of a counseling visit. In multivariate analysis, women 25–34 years (AOR: 0.53; 95% CI: 0.30–0.92) or ≥35 years (AOR: 0.49; 95% CI: 0.25–1.00) had lower odds of accessing contraceptive services than women 16–24 years. Women who reported a higher monthly income also had lower odds of accessing contraceptive services than women who were less wealthy (AOR: 0.68; 95% CI: 0.47–0.98). By contrast, multiparae were more likely to access reproductive health services within 90 days than women with no living children (AOR: 1.83; 95% CI: 1.17–2.88).

### 3.4. Predictors of Modern Contraceptive Use

In univariate analysis, women were less likely to report modern contraceptive use if ≥35 years, single, divorced, or widowed, with undisclosed HIV status, and if their partner's HIV status was unknown (Table 2). Conversely, women 25–34 years with one or more living children, those reporting higher monthly incomes, those with CD4^+^ cell counts ≥250 cells/uL or hemoglobin levels ≥8.1 g/dL, and those without a history of tuberculosis infection had increased odds of contraceptive use. In multivariate analysis, age, marital status, HIV status disclosure, parity, CD4^+^ cell count, and hemoglobin level remained associated with modern contraceptive use. Women ≥35 years old (AOR: 0.63; 95% CI: 0.52–0.77), single, divorced, or widowed women (AOR: 0.30; 95% CI: 0.27–0.34), and those for whom HIV status disclosure was unknown (AOR: 0.75; 95% CI: 0.64–0.87) had lower odds of using modern contraception. The odds of using modern contraception were higher among women with one or more living children (AOR: 1.47; 95% CI: 1.21–1.78), and among healthier women with CD4^+^ cell counts of 250–350 cells/uL (AOR: 1.18; 95% CI: 1.05–1.33) or ≥351 cells/uL (AOR: 1.23; 95% CI: 1.10–1.38) than among nullipara and those with low CD4^+^ cell counts. A similar association was observed for hemoglobin levels 8.1–9.9 g/dL (AOR: 1.56; 95% CI: 1.24–1.97) and ≥10.0 g/dL (AOR: 2.16; 95% CI: 1.72–2.71).

### 3.5. Predictors of Dual Method Use

In univariate analysis, women who were ≥35 years, single, divorced, or widowed and those who did not provide information regarding HIV status disclosure had a lower odds of dual method use (Table 3). Higher odds of dual method use were observed among women with one or more living children, those with CD4^+^ cell counts ≥351 cells/uL, and those who did not report a history of tuberculosis. In multivariate analysis, women ≥35 years (AOR: 0.51; 95% CI: 0.41–0.63) were less likely to report dual method use than those 16–24 years. Women who were single, divorced, or widowed were also less likely to use dual methods than married women (AOR: 0.75; 95% CI: 0.66–0.86). Conversely, women with one or more living children (AOR: 2.07; 95% CI: 1.59–2.70), those with CD4^+^ cell counts ≥351 cells/uL (AOR: 1.25; 95% CI: 1.09–1.45), and those with no prior history of tuberculosis infection (AOR: 1.17; 95% CI: 1.01–1.35) had higher odds of dual method use. 

## 4. Discussion

We successfully implemented a peer-led counseling intervention in ART clinics, focusing on dual family planning method use to prevent unintended pregnancies and STIs. Our initial projections were that 20,000 HIV-infected women would access care across the participating clinics during the 12-month implementation period. Our team of peer educators counseled 18,407 HIV-infected women enrolled in care during this period, demonstrating the feasibility of this approach in a low-resource, African setting. At their baseline counseling visit, nearly 60% of women reported use of a modern contraceptive method; the majority used solely condoms. Among contraceptive users, only 26.5% reported use of a highly effective modern contraceptive method. Dual method use was also low at 17.7%. 

Older, single women with lower CD4^+^ cell counts were less likely to be using modern contraception. These findings highlight a vulnerable population of HIV-infected women. As the health of women with lower CD4^+^ cell counts improves on ART and as unmarried women enter new sexual partnerships, both groups remain at risk for unintended pregnancy and STIs. Future reproductive health policy and programming should be expanded beyond a focus on married, healthy women [17]. 

We were disappointed at the low numbers of women who desired access to family planning services and were unable to obtain it in a timely fashion. We can only speculate as to the reasons for this. In Lusaka, family planning services are not integrated within HIV clinics, and most family planning clinics are only open to patients in the afternoons. A woman who has already spent much of her morning in an ART clinic is less likely to return to spend another half-day in a family planning clinic. Periodic stock-outs of reproductive health commodities may also limit women's access to family planning services. Additionally, we speculate that most messages in the community promote condom use over use of more effective modern contraception or dual method use. Providers' attitudes towards sexual and reproductive healthcare for HIV-infected patients [18, 19] and misconceptions regarding the safety of hormonal contraceptive methods among women on ART may also play a role in limiting HIV-infected women's access to family planning [20]. 

The strengths of our study include the successful implementation of a standardized and comprehensive reproductive health counseling intervention. This intervention was acceptable to both providers and patients. Using a peer educator model, we demonstrate that it is possible to reach a sizeable number of HIV-infected women attending public ART clinics. It also became evident through our intervention that strengthening reproductive health care requires stronger linkages between family planning clinics, STI services, and HIV screening and treatment [21], as well as improved community understanding. Our study had several limitations. Contraceptive method and condom use were self-reported and thus subject to bias. Additionally, we did not assess the impact of our counseling intervention on pregnancy rates.

This study confirms the findings of smaller Zambian clinical trials [12, 22, 23] and demonstrates that 40% of HIV-infected women on ART are not using any form of modern contraception. Among contraceptive users, less than 30% use highly effective modern methods and even fewer use dual methods. Our counseling intervention improved uptake of modern contraceptives and dual method use modestly. Demographic and Health Surveys have demonstrated that knowledge of modern contraception is high among Zambians [24]. Additional quantitative and qualitative research should address fertility desires and barriers to dual method use in this population, guiding future public health interventions. Male partner involvement through couples counseling should be investigated further [25]. 

Our findings also underscore important challenges faced by HIV-infected women attempting to access family planning services within the public health system. Unmet contraceptive need is as high as 40% among these women. Strengthening public-sector service provision, with the goal of ensuring that no woman is turned away from a family planning clinic empty handed, should be an urgent priority. Finally, integrating family planning service provision into HIV care and treatment clinics will likely improve access to reproductive health services [26]. 

## 5. Conclusions

We successfully demonstrate the feasibility of integrating comprehensive reproductive health counseling into HIV care and treatment. We confirm that nearly 60% of HIV-infected women receiving care within the Zambian public-sector report use of a modern contraceptive method. However, only 27% of these women use highly effective hormonal or long-term reversible methods, and even fewer women report dual method use. Additional efforts are needed to promote dual method use, particularly among older, unmarried women and those with more advanced HIV disease. These interventions should be coupled with health system changes that address critical bottlenecks in reproductive health service provision.

## Figures and Tables

**Table 1:** Baseline characteristics of HIV-infected women aged 16–50 years receiving antiretroviral treatment at 16 public-sector clinics in Lusaka, Zambia (November 2009–November 2010).

	Women with documented RH counseling		Women with no documented RH counseling visit		* P*-value
	*N*	Value	*N*	Value	
Age at 1st visit (years), median (IQR)	18,407	34.6 (29.9–39.7)	14,591	34.7 (29.5–40.9)	<0.001^++^
16–24	1,246	6.8%	1,263	8.7%	<0.001**
25–34	8,362	45.4%	6,232	42.7%	
≥35	8,799	47.8%	7,096	48.6%	

Marital status	14,180		11,135		
Married	8,523	60.1%	6,570	59.0%	0.076**
Single/divorced/widowed	5,657	39.9%	4,565	41.0%	

Education	16,910		13,441		
None	4,677	27.7%	3,785	28.2%	0.016**
Primary	5,603	33.1%	4,246	31.6%	
Secondary or more	6,630	39.2%	5,410	40.2%	

Number of living children, median (IQR)	14,385	2 (1–3)	11,373	2 (1–3)	0.061^++^
0	1,806	12.6%	1,505	13.2%	0.106**
≥1	12,579	87.4%	9,868	86.8%	

Monthly income	12,243		9,400		
<ZMK 200,000	4,532	37.0%	3,864	41.1%	<0.001**
≥ZMK 200,000	7,711	63.0%	5,536	58.9%	

CD4^+^ cell count (cells/uL), median (IQR)	18,231	394 (256–558)	14,419	357 (228–522)	<0.001^++^
<250	4,361	23.9%	4,187	29.0%	<0.001**
250–350	3,338	18.3%	2,819	19.6%	
≥351	10,532	57.8%	7,413	51.4%	

Hemoglobin (g/dL), median (IQR)	18,175	12.4 (11.3–13.4)	14,248	12.2 (11.0–13.1)	<0.001^++^
≤8.0	363	2.0%	482	3.4%	<0.001**
8.1–9.9	1,365	7.5%	1,276	9.0%	
≥10.0	16,447	90.5%	12,490	87.7%	

History or current tuberculosis	18,407		14,591		
Yes	3,441	18.7%	2,771	19.0%	0.493**
No	14,966	81.3%	11,820	81.0%	

Time on ART (days), median (IQR)	18,407	709 (262–1,302)	14,591	659 (245–1,180)	<0.001^++^

HIV status disclosure to partner	18,407		14,591		
Yes	7,823	42.5%	6,026	41.3%	0.028**
Unknown	10,584	57.5%	8,565	58.7%	

Partner's HIV status	18,407		14,591		
Negative	621	3.4%	456	3.1%	<0.001**
Positive	3,598	19.5%	2,599	17.8%	
Unknown	5,332	29.0%	4,362	29.9%	
Missing	8,856	48.1%	7,174	49.2%	

RH: reproductive health; IQR: interquartile range; ZMK: Zambian Kwacha; ART: antiretroviral therapy.

**Person's chi-square test; ^++^Wilcoxon rank sum test.

**Table 2:** Predictors of modern contraceptive use among HIV-infected women aged 16–50 years receiving antiretroviral treatment at 16 public-sector clinics in Lusaka, Zambia (November 2009–November 2010).

	Women using modern contraception	Women not using modern contraception	Crude OR (95% CI)	Adjusted OR (95% CI)
	*N* = 11,358	*N* = 7,049		
Age at 1st visit (years)				
16–24	736 (6.5%)	510 (7.2%)	1.00	1.00
25–34	5,747 (50.6%)	2,615 (37.1%)	1.52 (1.35–1.72)	1.05 (0.87–1.25)
≥35	4,875 (42.9%)	3,924 (55.7%)	0.86 (0.76–0.97)	0.63 (0.52–0.77)

Marital status				
Married	6,503 (72.9%)	2,020 (38.4%)	1.00	1.00
Single/divorced/widowed	2,414 (27.1%)	3,243 (61.6%)	0.23 (0.22–0.25)	0.30 (0.27–0.34)

Education				
None	2,862 (27.4%)	1,815 (28.2%)	1.00	1.00
Primary	3,493 (33.4%)	2,110 (32.7%)	1.05 (0.97–1.14)	1.13 (0.99–1.28)
Secondary or more	4,108 (39.3%)	2,522 (39.1%)	1.03 (0.96–1.12)	1.13 (1.00–1.27)

Number of living children				
0	1,041 (11.6%)	765 (14.2%)	1.00	1.00
≥1	7,947 (88.4%)	4,632 (85.8%)	1.26 (1.14–1.39)	1.47 (1.21–1.78)

Monthly income				
<ZMK 200,000	2,709 (35.9%)	1,823 (38.8%)	1.00	1.00
≥ZMK 200,000	4,835 (64.1%)	2,874 (61.2%)	1.13 (1.05–1.22)	1.03 (0.92–1.15)

CD4^+^ cell count (cells/uL)				
<250	2,473 (22.0%)	1,887 (27.1%)	1.00	1.00
250–350	2,042 (18.1%)	1,295 (18.6%)	1.20 (1.10–1.32)	1.18 (1.05–1.33)
≥351	6,746 (59.9%)	3,784 (54.3%)	1.36 (1.27–1.46)	1.23 (1.10–1.38)

Hemoglobin (g/dL)				
≤8.0	168 (1.5%)	195 (2.8%)	1.00	1.00
8.1–9.9	710 (6.3%)	655 (9.4%)	1.26 (1.00–1.59)	1.56 (1.24–1.97)
≥10.0	10,355 (92.2%)	6,090 (87.8%)	1.97 (1.60–2.43)	2.16 (1.72–2.71)

History or current tuberculosis				
Yes	1,999 (17.6%)	1,442 (20.5%)	1.00	1.00
No	9,359 (82.4%)	5,607 (79.5%)	1.20 (1.12–1.30)	1.06 (0.96–1.18)

HIV status disclosure to partner				
Yes	5,985 (52.7%)	1,838 (26.1%)	1.00	1.00
Unknown	5,373 (47.3%)	5,211 (73.9%)	0.32 (0.30–0.34)	0.75 (0.64–0.87)

Partner's HIV status				
Negative	476 (4.2%)	145 (2.1%)	1.00	1.00
Positive	2,700 (23.8%)	898 (12.7%)	0.92 (0.75–1.12)	0.76 (0.54–1.06)
Unknown	3,357 (29.6%)	1,975 (28.0%)	0.52 (0.43–0.63)	0.75 (0.56–0.99)
Missing	4,825 (42.5%)	4,031 (57.2%)	0.36 (0.30–0.44)	0.66 (0.48–0.89)

OR: odds ratio; 95% CI: 95% confidence interval.

**Table 3:** Predictors of dual method use among HIV-infected women aged 16–50 years receiving antiretroviral treatment at 16 public-sector clinics in Lusaka, Zambia (November 2009–November 2010).

	Women using dual methods	Women not using dual methods	Crude OR (95% CI)	Adjusted OR (95% CI)
	*N* = 2,247	*N* = 9,111		
Age at 1st visit (years)				
16–24	170 (7.6%)	566 (6.2%)	1.00	1.00
25–34	1,269 (56.5%)	4,478 (49.1%)	0.94 (0.79–1.13)	0.80 (0.62–1.04)
≥35	808 (36.0%)	4,067 (44.6%)	0.66 (0.55–0.80)	0.51 (0.41–0.63)

Marital status				
Married	1,407 (77.9%)	5,096 (71.7%)	1.00	1.00
Single/divorced/widowed	400 (22.1%)	2,014 (28.3%)	0.72 (0.64–0.81)	0.75 (0.66–0.86)

Education				
None	562 (27.0%)	2,300 (27.4%)	1.00	1.00
Primary	692 (33.2%)	2,801 (33.4%)	1.01 (0.89–1.14)	1.02 (0.86–1.21)
Secondary or more	830 (39.8%)	3,278 (39.1%)	1.04 (0.92–1.17)	1.06 (0.90–1.24)

Number of living children				
0	154 (8.4%)	887 (12.4%)	1.00	1.00
≥1	1,685 (91.6%)	6,262 (87.6%)	1.55 (1.30–1.85)	2.07 (1.59–2.70)

Monthly income				
<ZMK 200,000	538 (35.0%)	2,171 (36.1%)	1.00	1.00
≥ZMK 200,000	999 (65.0%)	3,836 (63.9%)	1.05 (0.93–1.18)	0.93 (0.82–1.07)

CD4^+^ cell count (cells/uL)				
<250	438 (19.7%)	2,035 (22.5%)	1.00	1.00
250–350	394 (17.7%)	1,648 (18.2%)	1.11 (0.96–1.29)	1.05 (0.87–1.26)
≥351	1,397 (62.7%)	5,349 (59.2%)	1.21 (1.08–1.37)	1.25 (1.09–1.45)

Hemoglobin (g/dL)				
≤8.0	28 (1.3%)	140 (1.6%)	1.00	1.00
8.1–9.9	120 (5.4%)	590 (6.5%)	1.02 (0.65–1.60)	1.01 (0.51–2.02)
≥10.0	2,076 (93.3%)	8,279 (91.9%)	1.25 (0.83–1.89)	1.31 (0.80–2.16)

History or current tuberculosis				
Yes	333 (14.8%)	1,666 (18.3%)	1.00	1.00
No	1,914 (85.2%)	7,445 (81.7%)	1.29 (1.13–1.46)	1.17 (1.01–1.35)

HIV status disclosure to partner				
Yes	1,291 (57.5%)	4,694 (51.5%)	1.00	1.00
Unknown	956 (42.5%)	4,417 (48.5%)	0.79 (0.72–0.86)	1.03 (0.85–1.25)

Partner's HIV status				
Negative	102 (4.5%)	374 (4.1%)	1.00	1.00
Positive	564 (25.1%)	2,136 (23.4%)	0.97 (0.76–1.23)	0.92 (0.69–1.22)
Unknown	721 (32.1%)	2,636 (28.9%)	1.00 (0.79–1.27)	1.03 (0.70–1.51)
Missing	860 (38.3%)	3,965 (43.5%)	0.80 (0.63–1.00)	0.89 (0.65–1.22)

OR: odds ratio; 95% CI: 95% confidence interval.
